# Reduced hemolytic complement activity in the classical pathway (CH50) is a risk factor for poor clinical outcomes of patients with infections: a retrospective analysis of health insurance claims in Japan

**DOI:** 10.3389/fimmu.2025.1601690

**Published:** 2025-06-05

**Authors:** Hiroyuki Koami, Yutaro Furukawa, Yuri Hirota, Akira Sasaki, Hirotaka Ogawa, Ayaka Matsuoka, Kota Shinada, Kento Nakayama, Ryota Sakurai, Sachiko Iwanaga, Takayuki Onohara, Shogo Narumi, Mayuko Koba, Hirotaka Mori, Yutaka Umemura, Kazuma Yamakawa, Kohji Okamoto, Yuichiro Sakamoto

**Affiliations:** ^1^ Department of Emergency and Critical Care Medicine, Saga University, Saga, Japan; ^2^ LOCOMOCO (Landmark of Clinical Observations in MicrOcirculation and Coagulation Outcomes) Study Group, Tokyo, Japan; ^3^ Department of Biostatistics, Hokkaido University, Hokkaido, Japan; ^4^ Division of Trauma and Surgical Critical Care, Osaka General Medical Center, Osaka, Japan; ^5^ Department of Emergency and Critical Care Medicine, Osaka Medical and Pharmaceutical University, Takatsuki, Osaka, Japan; ^6^ Department of Surgery, Kitakyushu City Yahata Hospital, Kitakyushu, Fukuoka, Japan

**Keywords:** CH50, infection, health insurance claims database, outcome, coagulopathy

## Abstract

**Purpose:**

To evaluate whether low CH50 (a comprehensive measure of hemolytic activity of the classical complement pathway) is associated with infection-related coagulopathy, organ dysfunction, and poor clinical outcomes.

**Methods:**

This was a retrospective study using Japanese health insurance claim data (2014-2023). Adult patients whose CH50 values were measured within one week of admission were included. We divided the patients into three groups based on the normal CH50 range: Low CH50 (< 25 U/mL; n=168), Normal CH50 (25 ≤, < 48 U/mL; n=1273), and High CH50 (48 ≤ U/mL; n=1285).

**Results:**

Of 2,726 patients who met the inclusion criteria, logistic regression models demonstrated that decreased CH50 is a significant predictor of 180-day mortality (OR: 0.98-0.99). Cumulative survival rates in the Low CH50 group at 28 days and 180 days were both unfavorable (both p < 0.0001, Log-rank test). CH50 was significantly inversely correlated with SOFA, SIC, ISTH-overt DIC, and JAAM-2 DIC scores, and was also correlated with C3 and C4 levels. Diminished CH50 may be particularly useful in diagnosing SIC (specificity; 79.2%) and excluding ISTH-overt DIC (sensitivity; 90.5%). Moreover, patients with low levels of both CH50 and C3 had an extremely high mortality rate (25.0%).

**Conclusion:**

Low CH50 after infection is not only significantly associated with multiple organ failure and coagulopathy but is also an independent risk factor for poor prognosis. Complement activation after infection may help to avert organ damage and to improve clinical outcomes.

## Introduction

Innate immunity serves as the first responder of the immune system. When foreign substances or microorganisms invade the body, the innate immune system recognizes them and triggers a rapid immune response that promotes phagocytosis and elimination of pathogens (1). The complement system, which is the central element in the innate immune system, is important in monitoring pathogens. When the complement cascade is activated, it triggers a sequential series of enzymatic reactions that help eliminate pathogens through bacterial opsonization, leukocyte recruitment and triggering, and pathogen destruction (2). More than 50 components comprise three major, trigger-dependent cascades that are modified by multiple regulators and receptors to maintain homeostasis against invaders (3). Three main types of complement cascades have been identified: classical (antibody-dependent), alternative (the mannose-binding lectin dependent), and lectin pathway (recognizing carbohydrate patterns on microbial surfaces). These pathways converge at C3 convertase, leading to the terminal pathway that forms membrane pores in target cells (2).

Recently, the concept “immunothrombosis” has been introduced, and infection induced complement activation not only amplifies the coagulation cascade through mechanisms such as platelet aggregation and thrombin generation, but also triggers immune defense (4). As an immunothrombosis, coagulation activation due to bacterial infections is acceptable in controlling the infection locally during the compensatory phase, but when this balance is disrupted, the disease can evolve into disseminated intravascular coagulation (DIC), causing thrombotic events in multiple organs (5). Previous studies have demonstrated that the prevalence of DIC is increasing in septic patients with complement activation, whereas marked complement activation has already been observed in severe sepsis and septic shock (6, 7). These observations suggest that complement activation is critical in controlling severity of infection and progression of coagulopathy.

CH50, an indicator of comprehensive hemolytic activity of the classical pathway (CP), is useful in diagnosing severity and predicting prognosis in lung cancer (8), heart failure (9), venous thromboembolism (10) and burn injuries (11). As a comprehensive measure of CP functionality, the CH50 test can identify when any component is reduced, missing, or inactive throughout the entire cascade, including those required for the cytolytic membrane attack complex (12, 13). On the other hand, several studies on complement activation in infectious diseases have reported that activation of anaphylatoxins, C3a and C5a, and the membrane attack complex (C5b-9) are useful predictors of disease severity, organ damage, and clinical outcomes; however, it is difficult to measure these markers in general clinical practice in Japan (6, 14, 15). Furthermore, studies on infection-induced changes in CH50 are few, making it difficult to provide feedback to clinical practice (7, 16–19).

In this study, we evaluated whether low CH50 values are associated with infection-related coagulopathy, organ dysfunctions, and poor clinical outcomes, utilizing big data from Japanese health insurance claims and various clinical data sources. We believe that this study is significant in that it provides a more comprehensive analysis by utilizing Japanese medical data, whereas previous clinical studies have faced limitations regarding the number of cases and measurement markers.

## Methods

### Data source

This study used commercially available, anonymized, insurance claim data from the Japan Medical Data Center (JMDC Inc.; Tokyo, Japan). The JMDC database includes a payer database with 17 million members registered since 2005, a personal health record database of 5.7 million members, a hospital database of 860 hospitals, a pharmacy database of 5700 pharmacies, and an electronic medical record database with 190 contracted medical institutions that represents approximately 14% of the total Japanese population, as of 2024 (JMDC Database. Available from: https://www.jmdc.co.jp/en/). When medical institutions claim medical treatment costs, diagnostic names of diseases are registered based in the International Classification of Diseases, 10^th^ revision (ICD-10) codes. We received an anonymized dataset from JMDC in September 2023. Then, we extracted and analyzed these data with necessary parameters using specific IDs.

### Study design and setting

This was a retrospective study that used the JMDC database. Patients who were admitted to hospitals due to infection and whose CH50 values were measured within one week of admission were included in the study (**Supplementary Table S1**). Patients younger than 18 years, patients with undiagnosed infections, patients with missing C3 or C4 data, patients with pregnancy, and patients with cardiac arrest on admission were excluded from the study. In addition, for patients with a history of multiple hospitalizations, only data from the earliest hospitalization were included. The present study was conducted and reported in accordance with the STROBE (Strengthening the Reporting of Observational Studies in Epidemiology) statement.

### Selection of participants

Enrolled patients were divided into three groups based on the initial CH50 value: Low CH50 group (< 25 U/mL), Normal CH50 (25 ≤, < 48 U/mL), and High CH50 (48 ≤ U/mL). This classification was based on the normal range of CH50 (25–48 U/mL).

### Data collection and definitions

In this study, the following factors were collected and analyzed: patient demographic factors (age, male, Charlson morbidity index, intensive care unit (ICU) admission, sepsis diagnosis), clinical indices related to organ damage (sequential organ failure assessment (SOFA) total score, every component of SOFA, such as respiratory, coagulation, liver, circulatory, central nervous system (CNS), and kidney, No of organ failure), coagulation/fibrinolysis abnormalities (sepsis-induced coagulopathy (SIC) score (20), SIC diagnosis, International society on thrombosis and hemostasis (ISTH) overt DIC score (21), ISTH overt DIC diagnosis, New Japanese association for acute medicine DIC score (JAAM-2 DIC score) (22), JAAM-2 DIC diagnosis, blood test findings (white blood cell (WBC), Neutrophils, Lymphocytes, Monocytes, hemoglobin (Hb), platelet count (Plt), prothrombin time-international normalized ratio (PT-INR), activated partial thromboplastin time (APTT), Fibrinogen, fibrin/fibrinogen degradation products (FDP), D-dimer, antithrombin (AT), albumin (Alb), C-reactive protein (CRP), lactate (Lac)), complement values (CH50, C3, C4), treatment modalities (ventilation, renal replacement therapy (RRT), use of vasopressor, heparin treatment, intravenous immunoglobulin (IVIG) therapy, AT therapy, recombinant thrombomodulin (rTM) therapy, serine protease inhibitors (SPI) treatment, history of blood transfusion during the first 7 days, each blood products transfusion), presence of bleeding/thrombotic complications and DIC during hospitalization, hospital length of stay and clinical outcomes. The recently proposed JAAM-2 score excludes the systemic inflammatory response syndrome (SIRS) component from the original JAAM DIC score, and the cutoff value for DIC diagnosis is a total of at least three points.

We based definitions of infection and sepsis on the Global Burden of Diseases, Injuries, and Risk Factors Study 2017 (23). All blood markers except serum complement values and clinical indices were evaluated on the day of admission. Normal values for complement C3 and C4, as well as CH50, were referenced to the normal ranges (C3; 86–160 mg/dL, C4; 17–45 mg/dL, CH50; 25–48 U/mL). The number of organ failures was counted for each organ component with a SOFA score ≥3. Diagnoses of SIC and ISTH-overt DIC were defined as scores of ≥4 and ≥5, respectively.

### Analysis

The median with interquartile range was used to represent continuous variables and the number with percent (%) was used to represent nominal variables. The Kruskal-Wallis test was employed to analyze continuous variables, and the Chi-square test was used for categorical/nominal data. The Steel-Dwass test was also used to analyze multiple comparisons and to set the significance level (alpha) to 0.05. We defined statistical significance of all analyses as *p* < 0.05. Logistic regression analysis was conducted to analyze whether CH50 is an independent predictor of mortality within 180 days. In addition, several models were created by increasing the number of factors extracted from the results of univariate analyses, and the odds ratios for CH50 were analyzed to assess the robustness of these models. Kaplan-Meier curves were constructed, and log-rank tests were performed to analyze 28-day, and 180-day survival based on the CH50 value. Correlation analysis was performed to evaluate factors correlated with CH50. Spearman’s rho was employed for correlation coefficients. As above, correlation coefficients between CH50 and C3, and CH50 and C4 were determined. Finally, receiver operating characteristic (ROC) analysis was performed to evaluate the diagnostic performance of CH50 for SIC, ISTH-overt DIC, and JAAM-2 DIC. Area under curve (AUC), cut-off values, sensitivity, and specificity were calculated. The cut-off value of CH50 was calculated using the Youden index. All statistical analyses were performed utilizing JMP^®^ Pro 16.1.0 software (SAS Institute, Cary, NC, USA).

## Results

**Figure 1:**
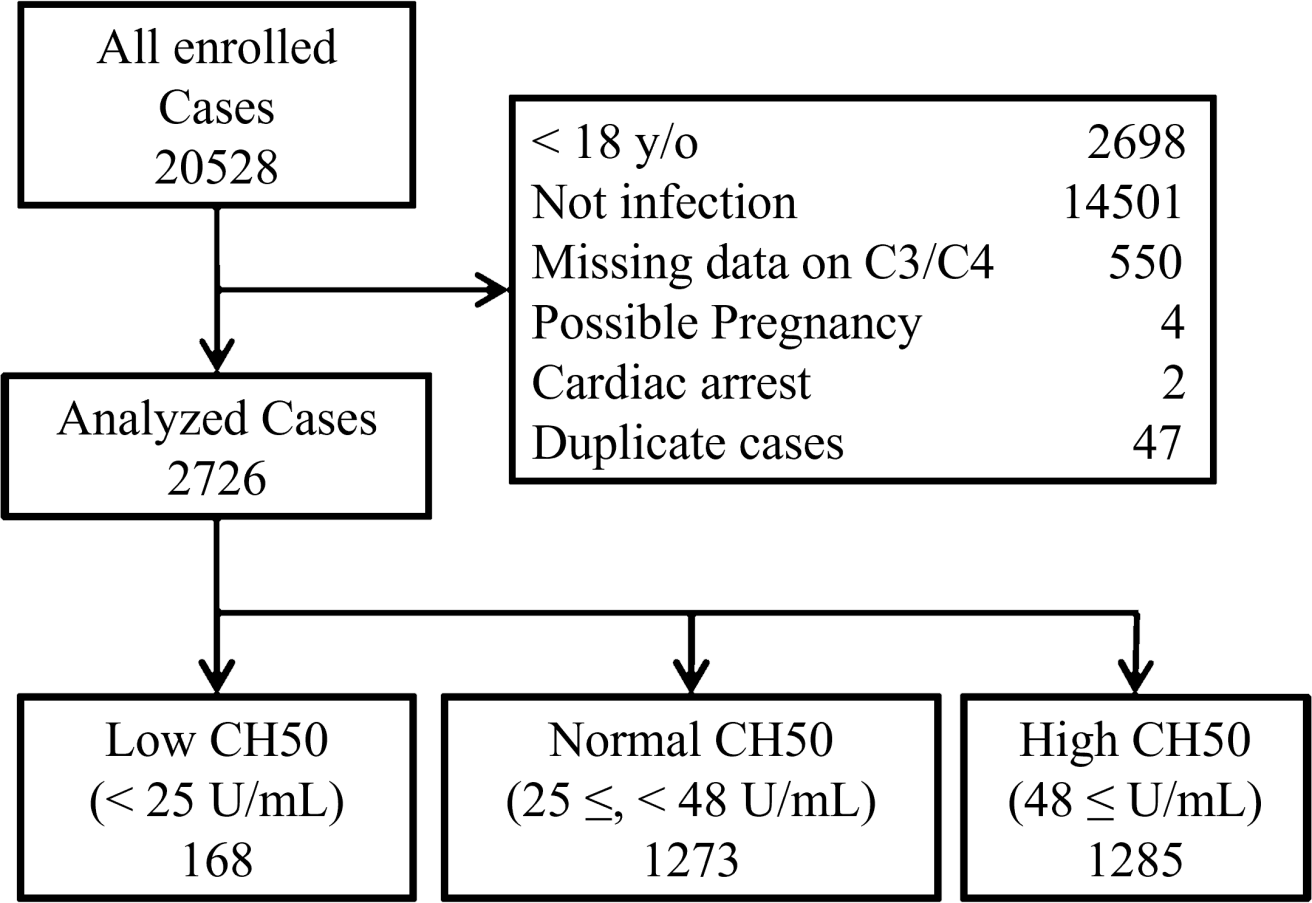
Study flow diagram.

From the original JMDC database, 20,528 patients fulfilled the inclusion criteria. After excluding 17,802 cases that met the exclusion criteria, 2,726 patients remained in the analysis of this study (**Figure 1**). Patients were categorized into the following three groups, according to their CH50 values: Low CH50 (< 25 U/mL; n = 168), Normal CH50 (25 ≤, < 48 U/mL; n = 1273), and High CH50 (48 ≤ U/mL; n = 1285).

Patient characteristics are shown in **Table 1**. The Low CH50 group was the oldest, with the highest Charlson index, and the highest rate of ICU admission. The percentage of sepsis was highest in the Low CH50 group.

**Table 1:** Patients characteristics in the analysis (n=2726).

Characteristic	Low CH50 (n=168)	Normal CH50 (n=1273)	High CH50 (n=1285)	*P* value
Age, y/o	77 [66, 87]	75 [60, 83]	71 [54, 81]	< 0.0001
Male, % (n)	53.0 (89)	53.3 (678)	53.8 (691)	0.96
Charlson morbidity index	6 [5, 9]	6 [4, 8]	5 [3, 7]	< 0.0001
ICU admission, % (n)	14.3 (24)	8.7 (111)	5.1 (65)	< 0.0001
Sepsis, % (n)	51.8 (87)	44.9 (571)	41.9 (538)	0.03

Continuous values are expressed as median [Q1, Q3]; *p <*0.05 was considered significant.

ICU, intensive care unit.

The median total SOFA score was the highest in the Low CH50 group (**Table 2**). A similar tendency was identified in terms of each component of the SOFA score such as coagulation, circulatory, and CNS. In addition, the median number of organ failures in the Low CH50 group was also the highest. On the other hand, the median SIC score, ISTH-overt DIC score and JAAM-2 DIC score were highest in the Low CH50 group. The percentage of patients meeting the SIC criteria was highest in the Low CH50 group. Statistically similar trends were also observed for the ISTH-overt DIC and the JAAM-2 DIC scores, although numbers of these cases were limited. Comparisons between CH50 groups showed statistically significant differences in all scores (**Supplementary Figure S1**). The median CH50 value in each group was 18 U/mL, 40 U/mL, and 55 U/mL, respectively (**Table 2**). Consistent with the change in CH50, C3 and C4 values showed a similar trend.

**Table 2:** Values of complement factors and treatments by CH50 levels (n=2726).

Characteristic	Low CH50 (n=168)	Normal CH50 (n=1273)	High CH50 (n=1285)	*P* value
SOFA	11 [5, 12]	6 [3, 8]	4 [2, 6]	< 0.0001
respiratory	2 [0, 3]	1 [0, 3]	0 [0, 2]	0.22
coagulation	1 [1, 2]	0 [0, 2]	0 [0, 1]	0.005
liver	0 [0, 1]	0 [0, 1]	0 [0, 1]	0.17
circulatory	3 [1, 4]	0 [0, 2]	0 [0, 0]	< 0.0001
CNS	2 [0, 3]	1 [0, 3]	0 [0, 2]	0.007
kidney	2 [0, 3]	1 [0, 2]	0 [0, 2]	0.13
No. of Organ Failure	2 [1, 3]	1 [0, 2]	0 [0, 1]	< 0.0001
SIC score	1 [0, 2]	0 [0, 1]	0 [0, 0]	< 0.0001
≥ 4, % (n)	9.1 (15/165)	2.6 (32/1223)	0.8 (10/1227)	< 0.0001
ISTH-overt DIC score	2 [0, 3]	1 [0, 2]	0 [0, 2]	0.001
≥ 5, % (n)	18.4 (7/38)	4.1 (12/293)	0.7 (2/283)	< 0.0001
JAAM-2 DIC score,	2 [1, 4]	1 [0, 2]	1 [0, 1]	< 0.0001
≥ 3, % (n)	37.7 (29/77)	22.2 (106/478)	16.8 (86/512)	< 0.0001
CH50, U/mL	18 [13, 22]	40 [34, 44]	55 [51, 63]	< 0.0001
C3, mg/dL	70 [52, 88]	102 [85, 120]	131 [113, 150]	< 0.0001
C4, mg/dL	17 [10, 23]	27 [22, 33]	34 [29, 41]	< 0.0001
Ventilation, % (n)	17.3 (29)	9.8 (125)	4.7 (60)	< 0.0001
RRT, % (n)	8.3 (14)	4.2 (53)	2.4 (31)	0.0002
Vasopressor, % (n)	22.0 (37)	11.6 (148)	7.7 (99)	< 0.0001
Heparin, % (n)	25.6 (43)	21.3 (271)	16.1 (207)	0.0003
IVIG, % (n)	1.2 (2)	2.7 (34)	2.3 (30)	0.48
AT, % (n)	3.0 (5)	1.5 (19)	0.2 (2)	< 0.0001
rTM, % (n)	3.6 (6)	1.0 (13)	0.9 (11)	0.006
SPI, % (n)	7.7 (13)	4.1 (52)	2.2 (28)	0.0002
Transfusion_7days, % (n)	14.9 (25)	9.4 (119)	4.0 (51)	< 0.0001
RBC_7days, U	0 [0, 0]	0 [0, 0]	0 [0, 0]	< 0.0001
FFP_7days, U	0 [0, 0]	0 [0, 0]	0 [0, 0]	< 0.0001
PC_7days, U	0 [0, 0]	0 [0, 0]	0 [0, 0]	< 0.0001

Continuous values are expressed as medians [Q1, Q3]; *p <*0.05 was considered significant.

SOFA, sequential organ failure assessment; CNS, central nervous system; SIC, sepsis induced coagulopathy; ISTH, international society on thrombosis and hemostasis; DIC, disseminated intravascular coagulation; JAAM, Japanese association for acute medicine; RRT, renal replacement therapy; IVIG, intravenous immunoglobulin; AT, antithrombin; rTM, recombinant thrombomodulin; SPI, synthetic protease inhibitor; RBC, red blood cell; FFP, fresh frozen plasma; PC, platelet concentrates.

According to treatment modalities, the Low CH50 group had the highest rates of ventilator management, renal replacement therapy, vasopressor use, heparin treatment, administration of antithrombin or thrombomodulin (which are used for DIC in Japan), and SPI use (**Table 2**). Furthermore, the Low CH50 group had the highest percentage of blood transfusions during the first week of hospitalization (approximately 15%), and statistically significant differences were confirmed for each transfusion product.

Other laboratory data are shown in **Supplementary Table S2**. White blood cell counts were similar in all groups, but the Low CH50 group had the largest neutrophil fraction and the smallest lymphocyte and monocyte fractions. Other blood count items, Hb and Plt, were lowest in the Low CH50 group. As for coagulation markers, the Low CH50 group showed the most prolonged PT-INR and APTT, and the highest D-dimer. A significant insufficiency of fibrinogen and a decrease in AT levels were also confirmed in the Low CH50 group. The Low CH50 group had the lowest Alb level and the least elevated CRP.

**Figure 2:**
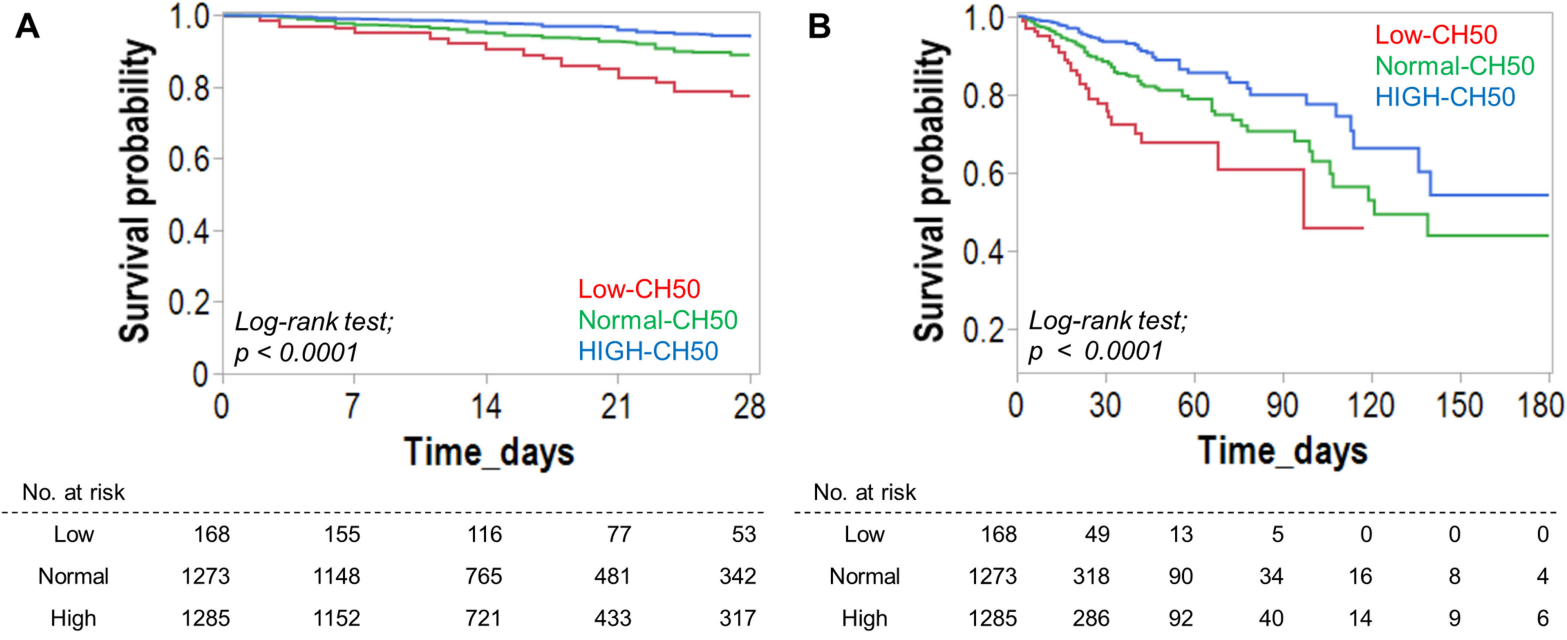
Effect of CH50 value during infection on survival. Survival analysis for 28-day **(A)** and 180-day **(B)** survival based on the degree of the CH50 value. Kaplan-Meier curves for 28-day **(A)** and 180-day **(B)** survival were created based on the CH50 level. The CH50 cut-off value was derived from the normal range. The Low-CH50 group showed a significantly lower survival rate.

**Table 3** shows complications after hospitalization and clinical outcomes. DIC was significantly more common in the Low CH50 group, while bleeding and thrombotic complications were not significantly different in any group. The longest hospital stays, as well as the highest mortality rate during the hospital stay, 28 days (15.5% vs 6.8% vs 3.2%, *p* < 0.0001), 90 days, and 180 days (19.6% vs 9.9% vs 5.1%, *p* < 0.0001) were also confirmed in the Low CH50 group. Overall cumulative survival rates for 28 days (**Figure 2A**) and 180 days (**Figure 2B**) were determined. At both time points, a decrease in CH50 was prognostically unfavorable by a statistically significant difference (both *p* < 0.0001, Log-rank test). Then, several logistic regression models were constructed using factors extracted from univariate analysis to determine whether a decrease in CH50 is a reliable predictor of 180-day mortality in patients with infections (**Table 4**). Lower CH50 was consistently a significant predictor of 180-day mortality in both models (OR: 0.978-0.987).

**Table 3:** Complications and clinical outcomes by levels of CH50 (n=2726).

Characteristic	Low CH50 (n=168)	Normal CH50 (n=1273)	High CH50 (n=1285)	*P* value
Complications after admission				
DIC, % (n)	4.2 (7)	1.9 (24)	1.3 (17)	0.03
Bleeding, % (n)	9.5 (16)	7.1 (90)	6.8 (87)	0.43
DVT, % (n)	5.4 (9)	4.5 (57)	5.5 (71)	0.47
PE, % (n)	2.4 (4)	5.3 (68)	6.2 (80)	0.11
Hp stay, days	19 [12, 31]	16 [10, 30]	15 [9, 27]	0.005
Mortality rates				
Hospital, % (n)	19.6 (33)	10.0 (127)	5.1 (66)	< 0.0001
28 days, % (n)	15.5 (26)	6.8 (86)	3.2 (41)	< 0.0001
90 days, % (n)	19.1 (32)	9.3 (118)	4.6 (59)	< 0.0001
180 days, % (n)	19.6 (33)	9.9 (126)	5.1 (65)	< 0.0001

Continuous values are expressed as median [Q1, Q3]; p <0.05 considered as significant.

DIC, disseminated intravascular coagulation; DVT, deep vein thrombosis; PE, pulmonary embolism; Hp, hospital.

**Table 4:** Multivariate analysis for mortality within 180 days (n=2726).

Characteristic	Univariate analysis			Multivariate analysis1			Multivariate analysis2			Multivariate analysis3		
	OR	95%CI	*P* value	OR	95%CI	*P* value	OR	95% CI	*P* value	OR	95%CI	*P* value
Age	1.05	1.04-1.07	< 0.0001	1.04	1.03-1.06	< 0.0001	1.06	1.04-1.07	< 0.0001	1.06	1.05-1.08	< 0.0001
Charlson morbidity index	1.18	1.13-1.22	< 0.0001	1.08	1.03-1.14	0.002	1.09	1.03-1.15	0.003	1.10	1.04-1.16	0.001
CH50	0.96	0.96-0.97	< 0.0001	0.98	0.97-0.99	< 0.0001	0.99	0.97-0.997	0.01	0.99	0.98-0.998	0.03
SIC score	1.67	1.49-1.86	< 0.0001	1.47	1.31-1.66	< 0.0001	1.32	1.16-1.51	< 0.0001	1.14	0.99-1.32	0.07
Ventilation	15.57	11.27-21.50	< 0.0001				14.42	9.94-20.92	< 0.0001	9.13	6.13-13.58	< 0.0001
Vasopressor	9.01	6.65-12.21	< 0.0001							4.09	2.72-6.14	< 0.0001

*p <*0.05 considered as significant.

SIC, sepsis induced coagulopathy; OR, odds ratio; CI, confidence interval.

Clinical scores and most markers were significantly correlated with CH50 (**Supplementary Table S3**). Therefore, to evaluate the predictive accuracy of CH50 for infection-induced coagulopathy or DIC, ROC curve analyses were performed (**Supplementary Figure S2**). The AUC was highest for ISTH-overt DIC (AUC: 0.764), with a sensitivity of 90.5% and specificity of 59.4% at the CH50 cutoff value of 44.0 U/mL. Based on the normal range of C3, C4, and CH50, we categorized all patients into three-by-three groups and evaluated the mortality rate of each population (**Supplementary Table S4**). Clinical outcomes were poorer in the low C3 (17.4%) and low C4 group (12.9%). Mortality rate was particularly high in patients with low CH50 and low C3 (25.0%), as well as in patients with low CH50 and normal C4 (22.6%).

## Discussion

In this retrospective study using Japanese big data from health insurance claims and clinical data sources, a low CH50 value was an independent risk factor for poor prognosis in patients with infection. CH50 was also useful in diagnosing organ damage and coagulopathy. Moreover, C3 and C4, which showed the same trend as CH50, suggested that in combination with CH50, it could further identify patient populations with poor clinical outcomes.

Limited evidence reported recently demonstrated that the relationship between CH50 and mortality in septic patients is inconclusive (16, 17). These studies had much smaller sample sizes, different definitions of sepsis, and mortality rates more than twice as high as the present study. In addition, a report analyzing 19 patients with bacterial meningitis demonstrated that complement values such as CH50, C3, and C4 of all patients were within the normal range (24). Considering the above, the findings of this study, which analyzed 2,726 patients with infectious diseases, are highly significant in that they reveal changes in complement markers that can be used clinically.

Decreased CH50 was also significantly associated with organ damage and coagulation abnormalities induced by infection. Not only the total SOFA score and the number of damaged organs, but also the organ dysfunction of each component of the SOFA score, including coagulation, circulation, and CNS were significantly associated with low CH50 value. This suggests that different organs have different susceptibilities to effects of infection-induced complement activation, like the CP. More interestingly, the mortality rate is lower in the high CH50 group than in the normal CH50 group. This means that high CH50 values associated with infection may be a pathophysiologically more congruent response in the acute phase of inflammatory response (25). Indeed, hypocomplementemia due to COVID-19 infection has been reported to be a risk for secondary bacterial infections through reduced opsonization and decreased hemolytic ability of MAC (12). In other words, complement activation is inadequate during this acute phase of infection, it can be assumed that the infection is poorly controlled, leading to organ failure and poor clinical outcomes.

In this study, strong positive correlations were demonstrated between both C3 and C4 with CH50. This confirms that CH50 primarily reflects comprehensive complement activation of the CP (26). In clinical studies of severe sepsis, C3 and C4 were reduced because C3 and C4 are degraded and activated to C3a and C4a, respectively (27). Furthermore, in a recently reported prospective study of severe sepsis and septic shock, the complement system was markedly activated, with significantly decreased C3 and C5 while significantly increased C3a and C5a, although CH50 was not measured (6). However, no correlation was found between complement activation and inflammatory markers, organ damage, or clinical outcomes. This suggests that in severe sepsis, remarkable complement activation has already developed and is not a useful predictor of clinical severity or outcome. Regarding the prognosis for each of the CH50 and C3 categories in this study, the mortality rate was higher in cases with low C3, and the worst mortality rate increased to 25.0% in the group with low CH50 and low C3. With respect to the combination of CH50 and C4, the mortality rate was higher than 10% in the normal or low CH50 and normal or low C4 groups. These differences are important in analyzing the impact of each complement pathway on complement activation in infectious diseases on clinical outcomes.

There are some limitations to the present study. First, although complement values can be routinely measured as a clinical test in Japan, it is unclear what the purpose of the measurement was in this study. In addition, complement values were measured within one week of admission, not necessarily on the day of admission; thus, it is difficult to define a causal relationship between actual complement values and pathophysiology of infection. Second, there is limited information on infectious diseases. For example, data on the source of infection, the need for drainage, the use of antimicrobial agents, and other treatment-related data were not analyzed in this study. In addition, it should be noted that the definition of sepsis in this study varies by age group. Fourth, information on indications and treatment details provided is limited in this study. Finally, the choice of specific laboratory tests to be performed is at the discretion of the physician in charge, leading to potential selection bias.

## Conclusions

Low CH50 in patients with infection was not only significantly associated with multiple organ failure and coagulopathy but was also an independent risk factor of poor prognosis. CH50 also showed a strong positive correlation with C3 and C4, and patients with low levels of both CH50 and C3 had an extremely high mortality rate. These findings suggest that infection-induced activation of the complement pathway is a normal acute-phase immune response, and that early recognition of these reactive disturbances may help to avoid organ damage and to improve clinical outcomes.

## Data Availability

The data analyzed in this study is subject to the following licenses/restrictions: Datasets generated and/or analyzed during the present study are not publicly available due to ethical restrictions associated with personal information, but anonymized data are available from the corresponding author upon request. Requests to access these datasets should be directed to Hiroyuki Koami, hkoami@cc.saga-u.ac.jp.
