# Supplementary material for: Reduced hemolytic complement activity in the classical pathway (CH50) is a risk factor for poor clinical outcomes of patients with infections: a retrospective analysis of health insurance claims in Japan
Source: Front Immunol. 2025 Jun 5;16:1601690. doi: 10.3389/fimmu.2025.1601690 (PMC12176544; doi:10.3389/fimmu.2025.1601690)
Supplement: Supplementary file 1 [file DataSheet1.docx]

**Supplemental Data**

**Reduced hemolytic complement activity in the classical pathway (CH50) is a risk factor for poor clinical outcomes of patients with infections: A retrospective analysis of health insurance claims in Japan.**

Hiroyuki Koami^1,2^*, Yutaro Furukawa^1^, Yuri Hirota^1^, Akira Sasaki^1^, Hirotaka Ogawa^1^, Ayaka Matsuoka^1^, Kota Shinada^1^, Kento Nakayama^1^, Ryota Sakurai^1^, Sachiko Iwanaga^1^, Takayuki Onohara^1^, Shogo Narumi^1^, Mayuko Koba^1^, Hirotaka Mori^2,3^, Yutaka Umemura^2,4^, Kazuma Yamakawa^2,5^, Kohji Okamoto^2,6^, Yuichiro Sakamoto^1,2^

***Correspondence:**

Hiroyuki Koami

hkoami@cc.saga-u.ac.jp

^1^Department of Emergency and Critical Care Medicine, Saga University, Saga, Japan.

^2^LOCOMOCO (Landmark Of Clinical Observations in MicrOcirculation and Coagulation Outcomes) study group

^3^Department of Biostatistics, Hokkaido University, Hokkaido, Japan.

^4^Division of Trauma and Surgical Critical Care, Osaka General Medical Center, Osaka, Japan.

^5^Department of Emergency and Critical Care Medicine, Osaka Medical and Pharmaceutical University, Takatsuki, Osaka, Japan

^6^Department of Surgery, Kitakyushu City Yahata Hospital, Kitakyushu, Fukuoka, Japan.

**Table of Contents:**

**Table S1. ICD-10 table……………………………………………………………………………….….…..….3**

**Fig. S1. Scores on organ failure and coagulopathy by the levels of CH50…………………………….…4**

**Table S2. Laboratory data by the levels of CH50……………………………………………….……….….5**

**Table S3. Correlation analysis of each clinical score or marker with CH50…………………………….6**

**Fig. S2. ROC analysis of CH50 for predicting SIC, ISTH-overt DIC, and JAAM DIC……………...…7**

**Table S4. Mortality rate within 180-day by the levels of the CH50 and C3 (A) or C4 (B)…………….8**

**Table S1. ICD-10 table**

| Group | Code type | Code |
| --- | --- | --- |
| Infection  (Implicit sepsis) | ICD 10 codes in the primary diagnosis or admission-precipitating diagnosis | A01-A02.0, A03-A09.9, A19-A20.3, A21-A21.3, A22-A22.2, A23-A24.0, A25-A26.0, A27-A28.1, A31-A32.12, A36-A39, A39.1-A39.3, A42-A42.2, A43-A46.0, A48-A49.9, A59-A59.9, A65-A65.0, A69-A69.1, A74, A74.8-A75.9, A77-A81.9,  A83-A96.9, A98-B00.59, B01-B10.89, B25-B27.99, B29.4,  B33-B34.9, B37-B37.6, B38-B50.9, B54-B55, B55.1-B55.9, B58-B60.8, B64, B67-B67.99, B91, B95-B99.9, G00-G08.0, G14-G14.6, H05.01-H05.039, H60.2-H60.23, H70.0-H70.009, I00, I02, I02.9, I26.01-I26.09, I26.90-I26.99, I33-I33.9,  I38-I39.9, I40.0-I40.9, I76, I96-I96.9, I98.1, J01-J06.9, J09-J22.9, J36-J36.0, J39.0-J39.1, J85-J86.9, K35-K37.9, K57-K57.93, K61-K61.4, K63.0-K63.1, K65-K65.9, K67.8, K75.0-K75.1, K75.3, K76.3, K77.0, K81.0, K81.2, K83.0, K95.01, K95.81, L02-L08.9, M00-M02.9, M86-M86.9, M89.6-M89.69,  N10-N10.9, N15.1-N15.9, N30-N30.91, N39.0, N41.0,  N41.2-N41.3, N45-N45.9, N70-N77.8, R78.81, T80.2-T80.29, T81.4, T82.6-T82.7, T83.5, T83.6, T84.5-T84.7, T85.7, T88.0, U04 |
| Infection  (Explicit sepsis) | ICD 10 codes in the primary diagnosis or admission-precipitating diagnosis | A02.1-A02.9, A20.7-A20.9, A21.7-A21.9, A22.7-A22.9,  A24.1-A24.9, A26.7-A26.9, A28.2-A28.9, A32.7-A32.9, A39.0, A39.4-A41.9, A42.7-A42.9, A50-A50.9, A54.86, B00.7-B00.9, B37.7-B37.9, N98.0, O03.0, O03.38, O03.5, O03.88, O04.5, O04.88, O07.38, O08.0, O08.83, O23-O23.93, O41.1-O41.93, O75.3, O85-O86.89, O88.3-O88.32, O91-O91.23, O98,  O98.2-O98.93, P00.2, P22-P23.9, P29.12, P29.81, P35-P37, P37.1-P39.9, R65.2-R65.21, R68.13 |
| *ICD 10* indicates International Classification of Diseases, Tenth Revision | | |

**
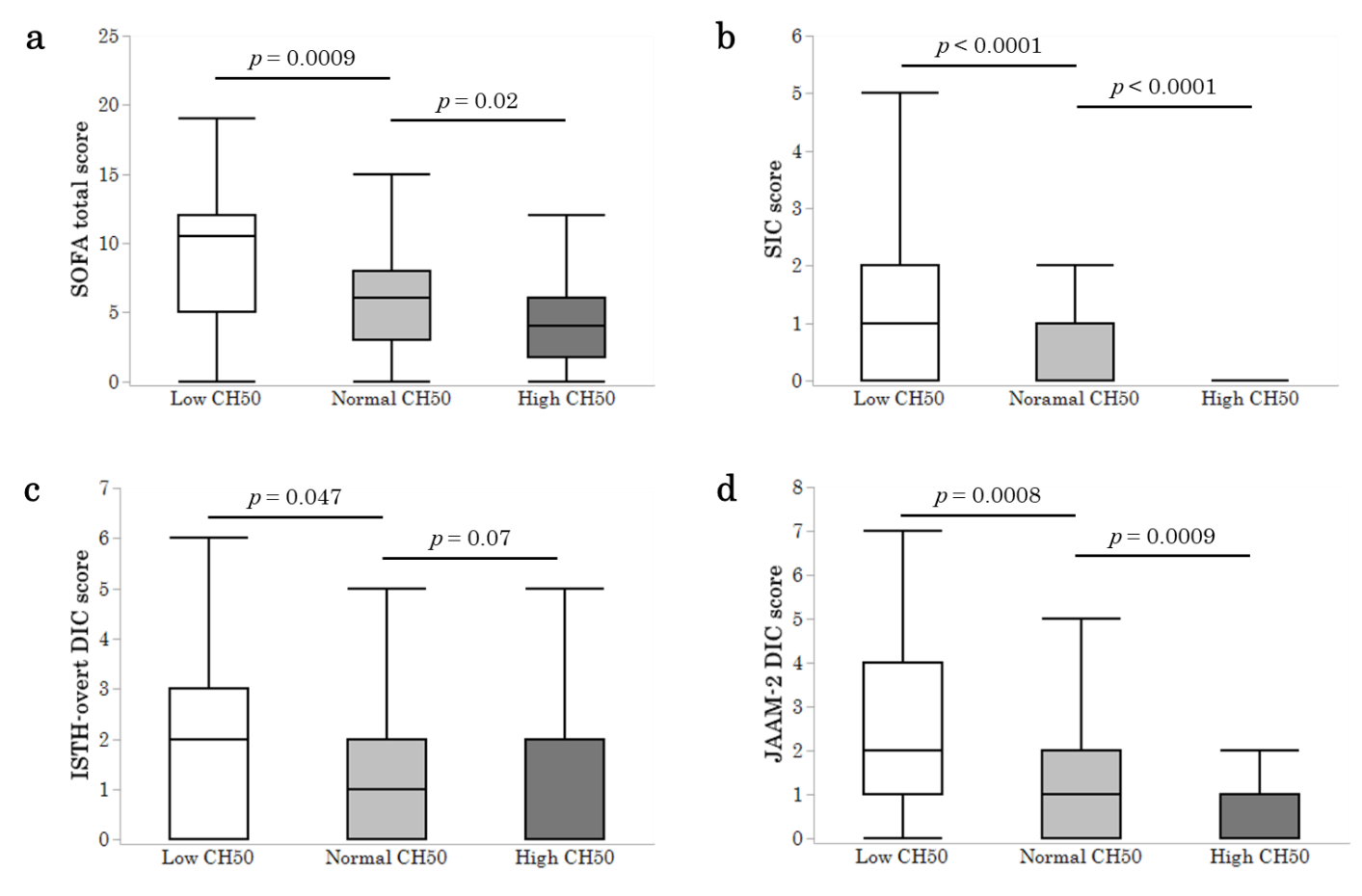
Fig. S1. Scores on organ failure and coagulopathy by the levels of CH50**

Low CH50 was significantly associated with higher SOFA score, higher SIC score, higher ISTH-overt DIC score, and higher JAAM-2 DIC score. Conversely, it is also interesting that High CH50 group was associated with lower scores for these scores compared to the Normal-CH50 group.

*p* <0.05 considered as significant

*SOFA* sequential organ failure assessment, *SIC* sepsis induced coagulopathy, *ISTH* international society on thrombosis and haemostasis, *DIC* disseminated intravascular coagulation, *JAAM* Japanese association for acute medicine

|  | Low CH50 (n=168) | Normal CH50 (n=1273) | High CH50 (n=1285) | *p* value |
| --- | --- | --- | --- | --- |
| WBC, /μL | 9620 [6280, 13745] | 9700 [6700, 13375] | 9500 [7100, 13000] | 1 |
| Neu, % | 83.3 [72.4, 89.7] | 81.0 [70.3, 88.5] | 79.1 [68.8, 86.0] | < 0.0001 |
| Lymph, % | 9.1 [4.5, 15.9] | 9.9 [5.6, 15.9] | 11.3 [7.0, 17.6] | < 0.0001 |
| Mono, % | 4.7 [3.0, 7.0] | 5.0 [3.3, 7.0] | 5.7 [4.0, 7.7] | < 0.0001 |
| Hb, g/dL | 11.3 [9.7, 12.7] | 11.9 [10.2, 13.5] | 12.5 [10.8, 13.8] | < 0.0001 |
| Plt, x 10^4^/μL | 16.4 [10.6, 24.8] | 20.1 [13.9, 26.7] | 22.9 [17.4, 30.4] | < 0.0001 |
| PT-INR | 1.16 [1.06, 1.35] | 1.12 [1.04, 1.23] | 1.10 [1.02, 1.19] | < 0.0001 |
| APTT, s | 32.6 [29.7, 34.6] | 30.6 [27.8, 35.4] | 30.0 [27.4, 34.2] | 0.01 |
| Fibrinogen, mg/dL | 368 [299, 541] | 456 [326, 602] | 547 [405, 700] | < 0.0001 |
| FDP, μg/mL | 11.6 [5.6, 32.2] | 8.3 [4.5, 17.0] | 7.9 [4.8, 17.5] | 0.31 |
| D-dimer, μg/mL | 4.64 [2.41, 10.55] | 3.50 [1.72, 7.78] | 2.90 [1.30, 8.20] | 0.003 |
| AT, % | 70.5 [52.7, 86.0] | 80.8 [66.8, 91.0] | 94.0 [79.2, 104.8] | < 0.0001 |
| Alb, g/dL | 2.9 [2.5, 3.4] | 3.3 [2.7, 3.8] | 3.4 [3.0, 3.8] | < 0.0001 |
| CRP, mg/dL | 6.8 [1.7, 16.6] | 8.4 [2.4, 15.5] | 9.5 [3.1, 17.4] | 0.002 |
| Lac, mg/dL | 11.7 [8.5, 18.4] | 13.3 [9.5, 21.8] | 14.5 [9.1, 20.6] | 0.84 |

**Table S2.** **Laboratory data by the levels of CH50**

Continuous values are expressed as median [Q1, Q3]; *p* <0.05 considered as significant

*WBC* white blood cells, *Neu* neutrophil, *Lymph* lymphocyte, *Mono* monocyte, *Hb* hemoglobin, *Plt* platelet, *PT-INR* prothrombin time-international normalized ratio, *APTT* activated partial thromboplastin time, *FDP* fibrin/fibrinogen degradation products, *AT* antithrombin, *Alb* albumin, *CRP* c-reactive protein, *Lac* lactate

**Table S3. Correlation analysis of each clinical score or marker with CH50**

| variable | vs variable | Spearman's rho | *p* value |
| --- | --- | --- | --- |
| CH50 | SOFA | -0.37 | < 0.0001 |
|  | SIC | -0.24 | < 0.0001 |
|  | ISTH-overt DIC | -0.18 | < 0.0001 |
|  | JAAM-2 DIC | -0.21 | < 0.0001 |
|  | C3 | 0.63 | < 0.0001 |
|  | C4 | 0.54 | < 0.0001 |
|  | WBC | -0.002 | 0.91 |
|  | CRP | 0.08 | 0.0001 |
|  | Plt | 0.20 | < 0.0001 |
|  | PT-INR | -0.13 | < 0.0001 |
|  | APTT | -0.09 | 0.002 |
|  | Fibrinogen | 0.23 | < 0.0001 |
|  | FDP | -0.07 | 0.10 |
|  | D-dimer | -0.13 | < 0.0001 |
|  | AT | 0.39 | < 0.0001 |
|  | Alb | 0.16 | < 0.0001 |
|  | Lac | -0.007 | 0.95 |

*p* <0.05 considered as significant

*SOFA* sequential organ failure assessment, *SIC* sepsis induced coagulopathy, *ISTH* international society on thrombosis and haemostasis, *DIC* disseminated intravascular coagulation, *JAAM* Japanese association for acute medicine, *WBC* white blood cells, *CRP* c-reactive protein, *Plt* platelet, *PT-INR* prothrombin time-international normalized ratio, *APTT* activated partial thromboplastin time, *FDP* fibrin/fibrinogen degradation products, *AT* antithrombin, *Alb* albumin, *Lac* lactate

**Fig. S2.** **ROC analysis of CH50 for predicting SIC, ISTH-overt DIC, and JAAM DIC**

**
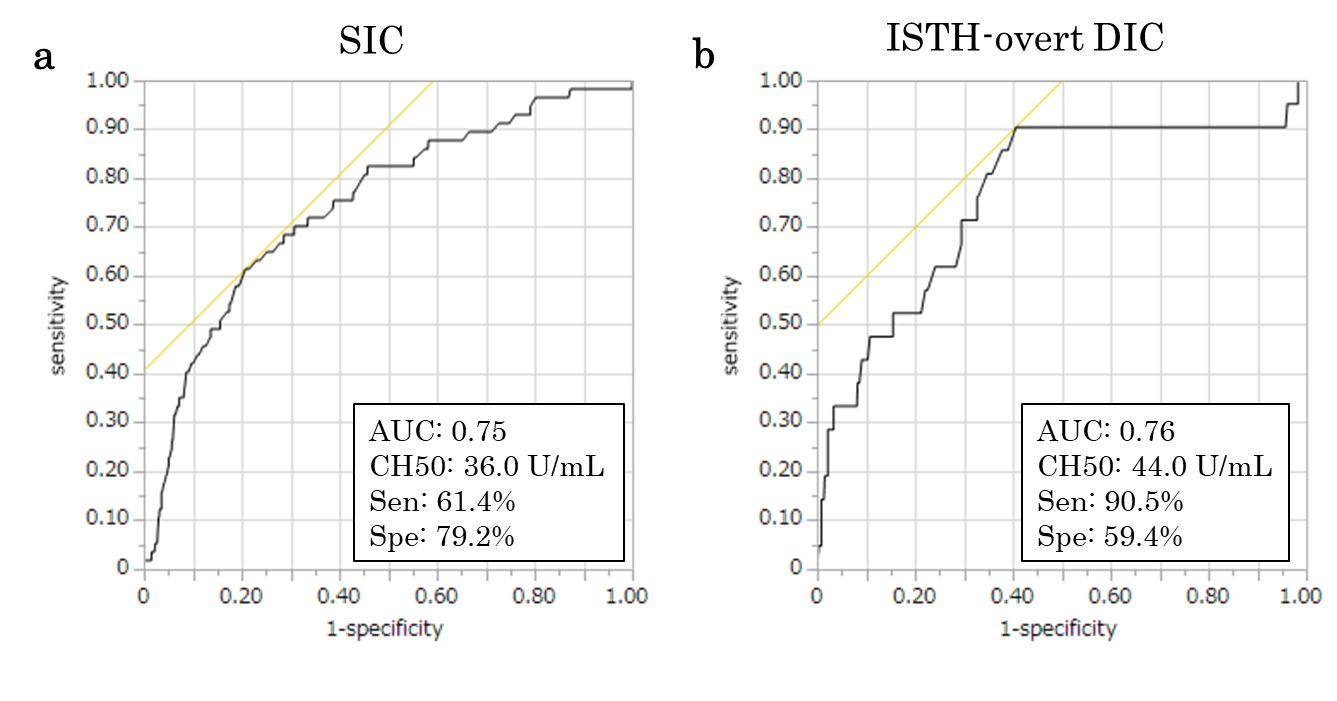
**

**
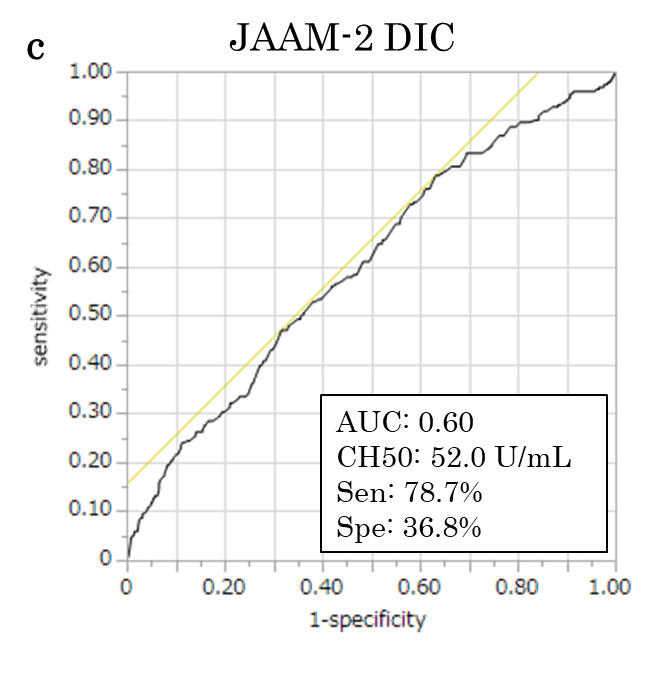
**

CH50 had particularly high sensitivity for diagnosis of SIC (a) and high specificity for diagnosis of ISTH-overt DIC (b) in patients with infection.

*SIC* sepsis induced coagulopathy, *AUC* area under the curve, *Sen* sensitivity, *Spe* specificity, *ISTH* international society on thrombosis and haemostasis, *DIC* disseminated intravascular coagulation, *JAAM* Japanese association for acute medicine

**Table S4. Mortality rate within 180-day by the levels of the CH50 and C3 (A) or C4 (B)**

| No. of Case | Low-C3  (< 86) | Normal-C3  (86 ≤,< 160) | High-C3  (160 ≤) | Total |
| --- | --- | --- | --- | --- |
| **Mortality** |  |  |  |  |
| Low-CH50  (< 25) | 120 | 45 | 3 | 168 |
|  | **25.0%** | **6.7%** | **0.0%** | **19.6%** |
| Normal-CH50  (25 ≤,< 48) | 327 | 920 | 26 | 1273 |
|  | **16.5%** | **7.7%** | **3.9%** | **9.9%** |
| High-CH50  (48 ≤) | 47 | 1020 | 218 | 1285 |
|  | **4.3%** | **5.7%** | **2.3%** | **5.1%** |
| Total | 494 | 1985 | 247 | 2726 |
|  | **17.4%** | **6.7%** | **2.4%** | **8.2%** |

**A**

| No. of Case | Low-C4  (< 17) | Normal-C4  (17 ≤,< 45) | High-C4  (45 ≤) | Total |
| --- | --- | --- | --- | --- |
| **Mortality** |  |  |  |  |
| Low-CH50  (< 25) | 83 | 84 | 1 | 168 |
|  | **16.9%** | **22.6%** | **0.0%** | **19.6%** |
| Normal-CH50  (25 ≤,< 48) | 112 | 1104 | 57 | 1273 |
|  | **11.6%** | **10.1%** | **1.8%** | **9.9%** |
| High-CH50  (48 ≤) | 15 | 1051 | 219 | 1285 |
|  | **0.0%** | **5.0%** | **5.5%** | **5.1%** |
| Total | 210 | 2239 | 277 | 2726 |
|  | **12.9%** | **8.2%** | **4.7%** | **8.2%** |

**B**

Chi-square test; *p* < 0.05

Chi-square test; *p* < 0.05

Chi-square test; *p* < 0.0001
